# Allergen immunotherapy for respiratory allergy: Quality appraisal of observational comparative effectiveness studies using the REal Life Evidence AssessmeNt Tool. An EAACI methodology committee analysis

**DOI:** 10.1002/clt2.12033

**Published:** 2021-06-14

**Authors:** Danilo Di Bona, Giovanni Paoletti, Derek K. Chu, Jack Pepys, Luigi Macchia, Enrico Heffler, Giorgio Walter Canonica

**Affiliations:** ^1^ Department of Emergency and Organ Transplantation School and Chair of Allergology and Clinical Immunology University of Bari Aldo Moro Italy; ^2^ Personalized Medicine, Asthma and Allergy Humanitas Clinical and Research Center IRCCS Rozzano Italy; ^3^ Department of Biomedical Sciences Humanitas University Pieve Emanuele Italy; ^4^ Departments of Medicine and Health Research Methods Evidence & Impact McMaster University; ^5^ The Research Institute of St. Joe’s Hamilton. Hamilton Ontario Canada

**Keywords:** AIT, RELEVANT, respiratory allergy, SCIT, SLIT

## Abstract

**Background:**

Observational comparative effectiveness studies in allergen immunotherapy (AIT) represent an important evidence source answering research questions that can be challenging to obtain from randomized controlled trials (RCTs), such as long‐term benefits of AIT, the effects on asthma prevention and the onset of new allergen sensitizations. However, observational studies are prone to several sources of bias, which limit their reliability.

The REal Life Evidence AssessmeNt Tool (RELEVANT) was recently developed to assist in quality appraisal of observational comparative research to enable identification of useful nonrandomized studies to be considered within guideline development.

**Objective:**

To systematically appraise the quality of published observational comparative AIT studies using RELEVANT.

**Methods:**

Observational studies comparing AIT to pharmacotherapy for respiratory allergies, assessing as outcome measures reduction of symptoms and/or medication use reduction, were retrieved by computerized bibliographic searches. According to RELEVANT, a failure to meet any one of primary items (background, design, measures, analysis, results, discussion/interpretation, and conflict of interest) represents a critical flaw, significantly undermining the validity of the study results.

**Results:**

The 14 studies identified supported the benefit of AIT in real‐life, which persists after treatment discontinuation. However, none of them met all the 7 primary RELEVANT criteria. The main defects were reported in the design (28.6% of studies), measures and analysis (64.3% of studies), and results (78.6% of studies) items, due to selection bias and lack of methods for adjusting controls. Half of the studies did not report on conflict of interest.

**Conclusion:**

There is a need for more robust observational research in AIT. RELEVANT appears as an easy‐to‐use and sensitive tool for quality appraisal in AIT studies.

## 1 INTRODUCTION

Allergen immunotherapy (AIT), administered by both the subcutaneous (SCIT) and sublingual (SLIT) routes, effectively treats allergic rhino‐conjunctivitis and asthma.^1^, ^2^, ^3^, ^4^, ^5^, ^6^ AIT’s benefit in reducing both the symptoms and the use of rescue medications lasts beyond the duration of the treatment and is therefore thought to be disease modifying.^7^, ^8^, ^9^, ^10^

Current guidelines recommend at least 3 years of therapy to obtain a sustained clinical benefit after AIT completion.^7^ This recommendation is mainly based on evidence from observational studies, since data from SLIT randomized controlled trials (RCTs) are limited.^8^, ^11^

However, evidence from observational studies is often ranked below that from RCTs in traditional evidence hierarchies, as they are prone to several sources of bias and most studies do not account for these.^12^, ^13^ Therefore, it is often difficult to assess whether AIT observational studies are of sufficient quality to be considered within the context of clinical guidelines, despite their importance in providing fundamental complementary information, such as treatment persistence, adherence, and long‐term benefit, that cannot, or are very challenging, to obtain from traditional RCT designs.

Recently, the Respiratory Effectiveness Group (REG) and European Academy of Allergy and Clinical Immunology (EAACI) joint Task Force developed the REal Life EVidence AssessmeNt Tool (RELEVANT) precisely in order to assist in quality appraisal of observational comparative effectiveness research.^14^, ^15^ The tool, like the Cochrane Collaboration’s Risk Of Bias In Non‐randomized Studies ‐ of Interventions (ROBINS‐I),^13^ is designed to identify evidence which are robust enough (i.e., low risk of bias) to inform clinical practice and to warrant consideration by guideline bodies.

Although RELEVANT was developed and has been validated for studies of asthma, it could, theoretically, also be applicable to general quality appraisal of observational comparative studies across other medical specialties.

The principal aim of this study was to systematically review observational studies on the effectiveness of AIT in treating of respiratory allergy as compared to standard therapy. This was done by RELEVANT in order to identify evidence of sufficient quality that can be integrated with the findings from RCTs, and thus provide a more complete picture on which to base clinical recommendations.

## 2 METHODS

This study is an EAACI position paper related to ROC, which commissioned the analysis, and its activities.

### 2.1 Data sources and searches

The primary sources of the reviewed studies were Medline, the Web of Science, and LILACS (inception to April 30, 2020) using a specific search strategy with the following medical subject headings: rhinitis, rhinosinusitis, rhinoconjunctivitis, conjunctivitis, asthma, specific immunotherapy, allergen immunotherapy, SIT, AIT, SLIT, SCIT, effectiveness, allergy, allergoid, new sensitizations, long term, follow‐up, real‐life, real‐world, retrospective, prospective, observational (see Supplementary file). The computer search was supplemented with manual searches of reference lists for review articles, primary studies, and abstracts from meetings. The search was limited to the English language literature.

### 2.2 Study selection

We required that studies: (i) were prospective or retrospective observational studies comparing subjects treated with AIT to subjects treated with standard pharmacotherapy who did not receive AIT; (ii) included monosensitized or polysensitized patients with allergic rhinitis/rhino‐conjunctivitis/rhino‐sinusitis and/or asthma with positive allergen‐specific skin prick tests, and/or elevated serum allergen‐specific IgE; and (iii) reported symptoms and/or medication use assessed by any measurement tool (e.g., symptom score, medication score, visual analogue score, etc.) as outcome measure of the treatment effect. Studies were excluded if they did not meet these criteria for study design or population, intervention, or outcomes of interest.

### 2.3 Data extraction and risk of bias assessment

Two separate reviewers (DDB, GP) independently extracted the study data. The accuracy of data extraction was confirmed by a third reviewer (EH). Disagreements were solved by consensus adjudication. We used the Real Life EVidence AssessmeNt Tool (RELEVANT) to evaluate the quality standards in the selected observational comparative effectiveness studies.^14^, ^15^ The tool guides systematic appraisal of the studies across seven quality domains (items): 1. Background; 2. Design; 3. Measures; 4. Analysis; 5. Results; 6. Discussion/Interpretation; 7. Conflict of interest (COI). For each quality domain, sub‐items are categorized as primary and secondary items. The seven items include a total of 11 primary and 10 secondary quality sub‐items. RELEVANT quality is defined as fulfillment of all 11 primary sub‐items: 1.1. Clearly stated research question; 2.1 Population defined; 2.2. Comparison groups defined and justified; 3.1. Exposure ‐e.g. treatment‐is clearly defined; 3.2. Primary outcomes defined; 4.1. Potential confounders are addressed; 4.2. Study groups are compared at baseline; 5.1. Results are clearly presented for all primary and secondary endpoints as well as confounders; 6.1. Results consistent with known information or if not, an explanation is provided; 6.2. The clinical relevance of the results is discussed; 7.1. Potential COI, including study funding, are stated.

Failure to meet any one primary item criterion is considered a potential fatal flaw in a study’s design, which may significantly undermine the validity of the results. Consequently, a study should only be eligible to inform guidelines development (or similar processes) if all primary items are satisfied. Thereafter, secondary items (not reported here) can be used to appraise non‐essential aspects of published studies to enable further characterization of their relative strengths and weaknesses.

## 3 RESULTS

**FIGURE 1:**
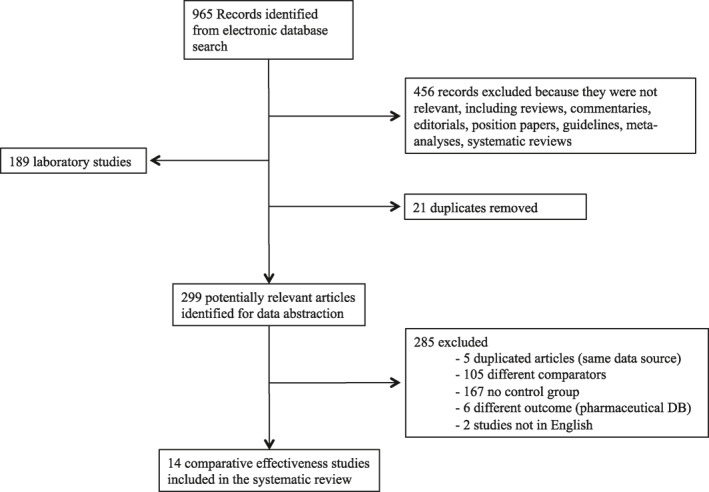
Flow diagram of AIT studies

Our search strategy identified 965 unique publications, including over 299 potentially relevant peer‐reviewed studies published from inception to April 30, 2020 (Figure 1). The full text of 21 studies was retrieved,^16^, ^17^, ^18^, ^19^, ^20^, ^21^, ^22^, ^23^, ^24^, ^25^, ^26^, ^27^, ^28^, ^29^, ^30^, ^31^, ^32^, ^33^, ^34^, ^35^, ^36^ of which 14 met the inclusion criteria (Figure 1).^16^, ^17^, ^18^, ^19^, ^20^, ^21^, ^22^, ^23^, ^24^, ^25^, ^26^, ^27^, ^28^, ^29^ We excluded the Jacobsen,^30^ Marogna,^31^ and Vesna^32^ studies because the participants were allocated to AIT or pharmacotherapy groups after randomization, the Wang,^33^ and Liu^34^ studies because the papers were not in English, the Jutel,^35^ and Devillier^36^ studies because they were based on data from longitudinal pharmacy databases identifying patients through data pertaining to drug prescriptions, and assessing effectiveness using drug prescription reduction, rather than a defined medication score tool, as surrogate measure of AIT benefit.

Table 1 shows descriptive data for the 14 systematically reviewed studies.^16^, ^17^, ^18^, ^19^, ^20^, ^21^, ^22^, ^23^, ^24^, ^25^, ^26^, ^27^, ^28^, ^29^ Four retrospective and 10 prospective cohort studies were identified (Table 1). In seven out of 14 studies patients were matched for a variable number of baseline characteristics.^16^, ^17^, ^18^, ^19^, ^22^, ^25^, ^29^ The majority of studies included a mixture of mono‐ and poly‐sensitized patients with rhinitis (Table 1). The five child,^16^, ^17^, ^18^, ^19^, ^20^ and nine adult studies^21^, ^22^, ^23^, ^24^, ^25^, ^26^, ^27^, ^28^, ^29^ included a total of 3629 patients (164 children, 3465 adults), of which 1973 (54.8%) were included in the only Bozek study.^28^ Eight of 14 studies were conducted in Italy,^17^, ^18^, ^19^, ^21^, ^22^, ^23^, ^24^, ^25^ one in Korea,^29^ and the remaining five in other European countries (Table 1).^16^, ^20^, ^26^, ^27^, ^28^ The treatment period ranged from 2 to 5 years and the post‐treatment follow‐up was from 0 to 12 years long. In some studies patient’s preference, or patient parents’ preference in pediatric studies, was taken into account for inclusion of participants in each group. The Dominicus study included as active cases a subgroup of subjects who underwent AIT, and as controls subjects who were screened but refused AIT in a previous RCT.^26^

**TABLE 1:** Characteristics of AIT comparative effectiveness studies

Study (yr)	Country	Study type	Groups	Participants over course of study	Female (%)	Mean Age, yrs (range)	Sensitization	Asthma (%)	Rhinitis (%)	Type of AIT	Outcome	Treatment duration (Yrs)^b^	Evaluation Period
CHILDREN													
Eng	Switzerland	P, M	AIT	14 → 13	23^a^	9.6 (5–16)^a^	Mono/Poly‐S	69	100^a^	SCIT	RSS, MS, SMS	3	6 years after EOT
2002			C	14 → 10	20^a^	8.8 (7–13)^a^		80	100^a^				
Di Rienzo	Italy	P	AIT	35 → 35	49	8 (3–17)	Mono‐Poly‐S	89^a^	100^a^	SLIT	ASS, MS	4 to 5	4 to 5 years after EOT
2003			C	25 → 25	48	9 (4–17)		92^a^	100^a^				
Acquistapace	Italy	P, M	AIT	90 → 81	28	11	Poly‐S	15	100^a^	SLIT	SS, MS	3	EOT
2009			C	81 → 81	27	12		24	100^a^				
De Castro	Italy	P, M	AIT	70 → 64	39	10.4 ± 3	Mono/Poly‐S	15^a^	86^a^	SLIT	RSS, ASS,	3	EOT
2013			C	70 → 63	47	10.7 ± 3		8^a^	91^a^		MS		
Djuric‐Filipovic	Serbia	P, M	AIT	34 → 34	–	13.2 ± 3.4	Mono/Poly‐S	–^a^	–^a^	SLIT	RSS, ASS,	2	EOT
2017			C	25 → 25	–			–^a^	–^a^		MS, LF, FeNO		
ADULTS													
Arena	Italy	P	AIT	79 → 71	45	22.4–35.5	Mono/poly‐S	39.1^a^	57.3^a^	SCITSLIT	Physician or patient opinion	3	EOT
2003			C	31 → 63									
Giovannini	Italy	P, M	AIT	15 → 15	47	24.2	Mono‐S	0	100	SCIT	SS, MS	3	EOT
2005			C	15 → 15	60	23.9							
Marogna	Italy	R, M	AIT	65 → 53	59	25.6	Mono‐S	‐^a^	–^a^	SLIT	RSS, ASS,	3	12 after EOT
2007			C	20 → 12	40	25.9		‐^a^	–^a^		MS, LF		
Marogna	Italy	R	AIT	69 → 57	–	18–28/55–65	Mono‐S	‐^a^	–^a^	SLIT	SMS, LF, drug use	3	EOT
2008			C	51 → 44	–	18–28/55–65		‐^a^	–^a^				
Milani	Italy	P, M	AIT	154 → 154	52	22	Mono/poly‐S	0	100^a^	SLIT	RSS, MS	2	EOT
2008			C	151 → 151	53	23		0	100^a^				
Dominicus	Germany	P	AIT	26	73	41.7 (24–63)	Mono/Poly‐S	n.r.	100^a^	SCIT	SMS, QoL	3	3 years after EOT
2012			C	13	54	33.9 (19–47)		n.r.	100^a^				
Drossaert	France	R, M	AIT	82 → 82	59	33.1 ± 11.6	Mono/Poly‐S	14	100^a^	SCIT	Symptoms, Medication use	3	EOT
2016			C	352 → 352	57	34.6 ± 12.7		12	100^a^				
Bozek	Poland	P, M	AIT	1006.967	50	25.1 ± 9.2	Mono‐S	21	98^a^	SCIT	RSS, ASS,	3	17 years after EOT
2017			C		49	19.7 ± 6.3		18	81^a^		MS		
Rhyou	Korea	R, M	AIT	56 → 48	51	43.4 ± 12.3	Mono/Poly‐S	100^a^	91.7	SCIT	ICS use reduction	3	EOT
2020			C	75 → 69	51	46.9 ± 16.1		100^a^	60.9				

Abbreviations: AIT, allergen immunotherapy; C, controls, subjects not treated with AIT; ASS, asthma symptom score; D. pt, *Dermatophagoides pteronissinus*; D. fa, *Dermatophagoides farinae*; EOT, end of treatment; HDM, House Dust Mite; ICS, inhaled corticosteroids; FU, follow‐up; LF, lung function; M, matched; Mono‐S, mono‐sensitized; MS, medication score; n.r., not reported; Poly‐S, poly‐sensitized; P, prospective; PNU, protein nitrogen units; QoL, Quality of Life; R, retrospective; RSS, rhinitis symptom score; SMS, symptom‐medication score; TU, therapeutic units; Yr, year.

→, patients analyzed at the end of the study.

^a^ Disease for which AIT was primarily indicated.

^b^ AIT acqueous extracts.

### 3.1 RELEVANT‐based quality assessment

**FIGURE 2:**
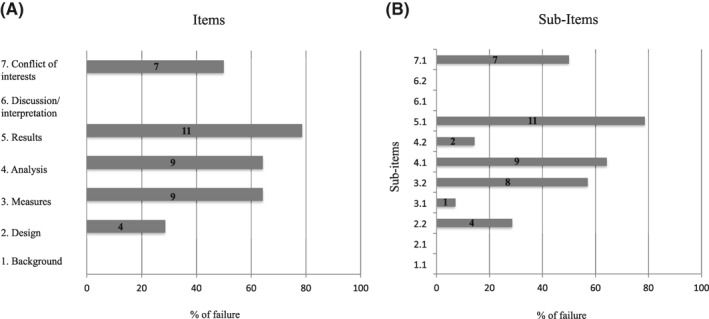
The numbers within the horizontal bars represent the number of studies reporting a failure in each specific item/sub‐item. Sub‐items: 1.1. Clearly stated research question; 2.1 Population defined; 2.2. Comparison groups defined and justified; 3.1. Exposure ‐e.g., treatment‐is clearly defined; 3.2. Primary outcomes defined; 4.1. Potential confounders are addressed; 4.2. Study groups are compared at baseline; 5.1. Results are clearly presented for all primary and secondary endpoints as well as confounders; 6.1. Results consistent with known information or if not, an explanation is provided; 6.2. The clinical relevance of the results is discussed; 7.1. Potential Conflict of interest, including study funding, are stated

The analysis started from the assessment of study quality based on the seven primary items (11 sub‐items) which are critical for enabling a study to be deemed of sufficient quality to be considered for guideline development. The percentage of specific criteria failure for the 14 studies is reported in Figure 2.

1. Background. We found that “The research question was clearly stated” in all the analyzed studies. Generally, the effectiveness of AIT, assessed as (i) symptom or drug use reduction during or at the end of the recommended 3‐years course, (ii) and/or the duration over time of AIT benefit after the completion of the treatment course, were the main research questions. Other outcomes, such as the development of new allergen sensitizations,^17^, ^18^, ^25^, ^26^ or lung function improvement were also analyzed in some studies.^20^, ^24^ One study assessed as measure of efficacy the AIT the inhaled corticosteroid‐sparing effect in patients with asthma (considered as asthma medication score).^29^ There was no failure in the fulfilment of this item (Figure 1).

2. Design. This item was fulfilled in nine studies.^16^, ^17^, ^22^, ^23^, ^24^, ^26^, ^27^, ^28^, ^29^ The “Population was defined” in all studies (sub‐item 2.1). Regarding the comparison groups (sub‐item 2.2), Five studies did not clarify the criterion used for patient’s allocation in each respective group.^18^, ^19^, ^20^, ^21^, ^25^ The other studies declared that the allocation was related to the patient’s or the parent’s choice or was based on clinical reason, according to guidelines (patients with a severe disease, with chronic exposure to allergens, such as animal dander, who were willing to reduce drug use). No study used a historical control.

3. Measures. One study failed to clearly define which AIT vaccine or vaccines was used in the treatment group (exposure: sub‐item 3.1).^29^ In particular, the authors did not report which route of AIT they used (SCIT or SLIT), the AIT allergen extract (e.g., pollens or house dust mites), vaccine formulation (e.g., native‐conjugated or allergoid) and the manufacturer.^29^

Eight studies did not report which was the primary outcome (sub‐item 3.2).^17^, ^19^, ^20^, ^21^, ^23^, ^24^, ^26^, ^28^ They analyzed different outcomes, including symptom and medication burden, but the primary outcome was not indicated in the Method section.

4. Analysis. We considered a list of potential confounders to assess if they were addressed by the investigators of the included studies (sub‐item 4.1): disease duration; severity of the disease at baseline; presence and severity of co‐morbidities; type of allergen (seasonal, perennial); mono‐ or poly‐sensitization; exposure to animal danders or molds; socio‐economic status. Nine out of the 14 studies did not fully address potential confounders.^16^, ^17^, ^18^, ^20^, ^21^, ^22^, ^26^, ^27^, ^28^, ^29^ The main defect of the studies was the absence of appropriate statistical analyses and interpretation to account for possible confounders, imbalance of prognostic factors, and/or treatment effect modifiers.

In all studies, except one,^21^ groups were compared at baseline (sub‐item 4.2). However, the number and importance of characteristics considered for the comparison varied greatly across the studies.

5. Results. Results for primary and secondary endpoints, as well as possible confounders are clearly presented only in three out of 14 studies.^19^, ^25^, ^29^ In particular, the importance of possible confounders was not considered.

6. Discussion/interpretation. Generally, the results of all the observational studies analyzed confirmed findings from previous RCTs that AIT is effective in the pragmatic settings (“real‐life”) (sub‐item 6.1). However, additional information relative to RCTs was provided. Five studies reported on the persistence of AIT benefit over long post‐treatment follow‐up, providing evidence supporting a prolonged disease‐modifying effect (Table 2). The effect of AIT in the onset of new allergen sensitizations, asthma development, patient’s quality of life, work and school performance, lung function (FEV1) or inflammation (FeNO) were also shown by some studies (Table 2).^16^, ^17^, ^18^, ^20^, ^21^, ^23^, ^25^, ^26^

**TABLE 2:** Summary table of literature analysis

Reference	Statement	Similar evidence from RCTs	Additional data relative to RCTs
Acquistapace	A 3‐years SLIT course is effective in reducing rhinitis symptoms (RSS) medications (MS), and occurrence new sensitizations.	Yes	New sensitizations
Arena	A 3‐years SLIT/SCIT course effective in reducing rhinitis symptoms and drug consumption according to physician’s and patient’s opinion, in increasing patient’s satisfaction, and reducing school and work days lost.	Yes	Patient’s satisfaction, working and school days lost
Bozek	AIT effectiveness persists after discontinuation (long‐term follow‐up, 20 years).	No	Effect after discontinuation
De Castro	A 3‐years SLIT course is effective in reducing rhinitis and asthma symptoms (RSS, ASS) and medications (MS).	Yes	No
Di Rienzo	A 3‐years SLIT course is effective in reducing asthma development, asthma symptoms (ASS) and new sensitizations (MS). The benefit persists after discontinuation (long‐term follow‐up, 4 to 5 years)	No	Effect after discontinuation, asthma development, new sensitizations
Djuric‐Filipovic	A 2‐years course of SLIT is effective in reducing rhinitis symptoms (RSS), asthma symptoms (ASS), medications (MS)	Yes	FeNO, FEV_1_
Dominicus	AIT effectiveness persists 3 years after discontinuation (in comparison with AIT untreated controls).	No	Effect after discontinuation, new sensitization, QoL
Drossaert	A 3‐years SLIT course is effective in reducing symptoms and medication use, as resulted by questionnaires (retrospective assessment).	Yes	No
Eng	AIT effectiveness in reducing symptoms and medication use persists after discontinuation (12 years after discontinuation).	No	New sensitization, long‐term FU after discontinuation
Giovannini	A 3‐years SLIT course is effective in reducing rhinitis symptoms (RSS) medications (MS)	Yes	No
Marogna ‘07	AIT effectiveness in reducing symptoms and medication use persists after discontinuation (up to 8 years after discontinuation).	No	Effect after discontinuation
Marogna ‘08	AIT effectiveness in reducing symptoms and medication after 3‐years treatment	Yes	Lung function
Milani	A 3‐years SLIT course is effective in reducing symptoms and medication use	Yes	New sensitizations
Rhyou	>1 year AIT course reduces ICS in asthmatic patients at 3 years from start of AIT	No	Effect after discontinuation

Abbreviations: ASS, asthma symptom score; FeNO, fractional exhaled nitric oxide; FEV_1_, forced expiratory volume in 1 s; MS, medication score; QoL, Quality of Life; RSS, rhinitis symptom score.

7. COI. A statement on COI was present in 7 studies. ^18^, ^19^, ^21^, ^25^, ^27^, ^28^, ^29^ ^.^ Four studies were authored by employees of or funded by pharmaceutical companies,^18^, ^21^, ^22^, ^25^ whereas the authors of 4 studies declared no COI.^19^, ^27^, ^28^, ^29^ In seven studies (50%) a COI’s disclosure was missing.^16^, ^17^, ^20^, ^22^, ^23^, ^24^, ^26^

## 4 DISCUSSION

We have established RELEVANT as a tool to distinguish high‐ and low‐quality comparative effectiveness studies by systematic review. Most observational “Real life” studies confirmed the principal findings of RCTs evaluating AIT. However, observational studies supported that AIT’s benefit persists years after AIT discontinuation, a finding which is challenging for most traditional RCTs to adequately evaluate.

The magnitude of this benefit is difficult to estimate, owing to different scales and scoring systems used in the studies, hindering the possibility of a quantitative synthesis. However, a consistent positive effect was reported (Table 2), and consistency is a useful criterion for making causal inferences from observational studies, as long as the consistency is not produced by a pervasive systematic confounder, such as a selection bias, or by a set of systematic biases that together produce a consistent bias in the same direction across studies.

Unfortunately, none of the 14 AIT comparative effectiveness studies included in this analysis meet all the 11 RELEVANT primary sub‐items, and are therefore deemed of insufficient quality to be eligible to robustly inform guidelines development.

As expected, selection bias was the most important limitation to internal validity of the studies, hindering the ability to make valid causal inferences for AIT effectiveness (Figure 2; domains 2 and 4). About 80% of the studies insufficiently recognized and controlled pre‐existing characteristics of the groups being compared, which could potentially lead to distinct prognoses (domains 2, 4 and 5). Most studies did not report sufficient details on baseline population characteristics. Only a couple of studies tried to control for this problem, making groups more comparable based on matching by other baseline characteristics, such as patients’ sensitization status (mono‐ or poly‐sensitization), allergy duration, comorbidities, disease severity at baseline, persistent or seasonal disease. Adjusting controls by these characteristics might have mitigated the role of confounders. For example, patients with more severe presenting symptoms may be more likely to get selected for an intervention (i.e., confounding by indication), so it should be important to match patients and controls for disease severity. Thus, when the choice was based on patient’s preference, we assume that a patient who refused AIT was comparable to those who accepted it in terms of disease severity. However, when the AIT was based on recommendations from the guidelines (more severe disease, side effects with standard therapy, chronic allergen exposure, e.g. animal dander, or willingness to reduce drug use by the patients), it is more likely that there are greater differences between patients and controls.^7^, ^37^, ^38^

The attention paid to selection bias in RELEVANT is highlighted by the impact of confounders in three different primary items: Design (primary item #2), Analysis (primary item #4) and Results (primary item #5). This caused some uncertainty when we rated the studies, owing to the fact that neglecting to account for confounders duplicates the negative rating. For example, if confounders are not taken into account in the study design (matching treatment and controls) or adjusted for by statistical analysis (sub‐item 4.1), as a consequence they will not be clearly presented in the result section (item 5) either, and thus leading to a negative rate for two or three items implying confounders. However, considering that a failure even in a single item is considered a fatal flaw by RELEVANT, no difference in the final judgment may arise due to a doubtful interpretation of this part of the tool.

Attrition bias may also have affected the results. This was not accounted for in most studies. In particular, it was completely ignored by the authors of the largest study, which did not report the rate or reasons of patients lost‐to‐follow up,^28^ potentially changing the characteristics of the groups, irrespective of the exposure or intervention. Methods to address missing data were also absent.

Another defect observed in nine studies was the lack of definition of the exposure (sub‐item #3.1),^29^ or the primary outcome (item #3.2). Furthermore, some studies used newly created tools to assess the outcome, or ad hoc modification of an existing measurement instrument, tool, or scale, without any supporting evidence of its validity and reliability. This generated unreliable conclusions and interpretation problems on the extent of the difference in treatment effectiveness across different outcomes.

Regarding COI, despite a general consensus favoring disclosure, a disclosure statement was present in only seven studies, four of which declaring no COI. This was independent of publication year.

Although disclosure only reveals the possibility of bias, without any guidance to resolve it,^39^ disclosure in observational studies is important to enable clinicians to evaluate research reports in the context of a clear description of COI and form their own opinion on the reliability of the results. On the other hand, the majority of RCTs are completely funded by industry, and as a result a substantial proportion of primary evidence is being produced by researchers who hold COI. However, RCTs registered in public databases (e.g., ClinicalTrials.gov) and enrolling large number of patients tend to have a lower risk of bias, relative to observational studies similarly disclosing COI or funded by industry, owing to randomization and blinding.

Nonetheless, RELEVANT considers as a fatal flow only the lack of COI disclosure, not a disclosed COI, despite the fact that it represents an important source of bias, which should be accounted for in the context of systematic revision, like any other confounder which can be measured and statistically accounted for when synthetizing the evidence.

The “background” (item #1), and discussion/interpretation (item# 6) items were fulfilled in all the studies, which highlights the strength of observational studies, potentially being able to provide additional information complementary to RCTs. These studies, while confirming the treatment benefit shown by RCTs, provide evidence on effectiveness over a 3‐ to 5‐years treatment, and the persistence of AIT effect after treatment completion. Unfortunately, important information such as adherence to AIT is lacking.

### 4.1 Strengths and limitations

This is the first time that a tool specifically designed for the appraisal of observational asthma research is used in AIT research. Notably, the proportion of RELEVANT failed items reported in our analysis is comparable with that observed in asthma studies,^15^ which showed no failure in the item #1 (Background) and only 5% studies with a failure in item #6 (Discussion and Interpretation). This consistency may suggest that the tool is suitable for the use in fields other than asthma, being sensitive to the main limitation of the real‐life studies.

This analysis has some limitations. Regarding the specific search strategy, we encountered some difficulties since the outcome was not clear in the title and in the abstract of the retrieved articles. Furthermore, the definition of AIT changed over the years, and we used different search term to retrieve as many studies as possible. Despite the possibility that some studies were overlooked, considering the general results, we are confident that they are not likely to have substantially changed the findings of this analysis.

Some uncertainty in interpretation of specific RELEVANT items emerged during the review process. This was probably due to the absence of a user guide, as for other tools, such as GRADE or ROBINS‐I, which would have made the tool simpler, reducing potential inter‐rater variability.^12^, ^13^ ^.^

Finally, it appears that RELEVANT assessments are influenced by the quality of reporting of research as much as the inherent quality of the study itself, as acknowledged by the EAACI‐REG Task Force members. This may result in an underestimation of the quality of the study analyzed. Therefore, a comparison with different established tools such as ROBINS‐I, which separates risk of bias from methodological quality and reporting quality, and GRADE, which systematically evaluates the quality of an entire body of evidence, is necessary in order to inform how to best determine the strength of recommendations on AIT.^12^, ^13^

In conclusion, this analysis based on RELEVANT allowed us to identify the main defects of comparative effectiveness research on AIT available to date. Based on the results of this analysis, we found a general lack of high‐quality real‐life effectiveness observational research.

As a consequence, we recommend that future studies should pay close attention to methods for adjusting confounders, clearly define primary outcomes, population and comparison groups, and state potential COI. This will help in providing reliable information that can hardly be obtained from RCTs, such as the duration of benefit after AIT discontinuation, treatment persistence, and adherence. In light of this, establishing AIT registries, with the aim of collecting data in a cohesive way, using standardized protocols will provide an essential source of RWE to promote evidence‐based research and quality improvement in study design and clinical decision‐making.^40^

## CONFLICT OF INTEREST

The Authors declare they have no conflict of interest.

## AUTHOR CONTRIBUTION

Danilo Di Bona, Giovanni Paoletti, and Giorgio Walter Canonica developed the concept of this study. Danilo Di Bona and Giovanni Paoletti, collected and analyzed the data for the study. The first draft of the manuscript was written by Danilo Di Bona and thoroughly revised by Jack Pepys, Derek K. Chu and Giorgio Walter Canonica. Enrico Heffler and Luigi Macchia, provided critical revision of the manuscript. Danilo Di Bona and Giovanni Paoletti have contributed equally.

## Supporting information

Supporting Information 1

Supporting Information 2

Supporting Information 3
