# Supplementary material for: Allergen immunotherapy for respiratory allergy: Quality appraisal of observational comparative effectiveness studies using the REal Life Evidence AssessmeNt Tool. An EAACI methodology committee analysis
Source: Clin Transl Allergy. 2021 Jun 14;11(4):e12033. doi: 10.1002/clt2.12033 (PMC8203181; doi:10.1002/clt2.12033)
Supplement: Supplementary file 1 — Supporting Information 1 [file CLT2-11-e12033-s001.docx]

ALLERGEN IMMUNOTHERAPY FOR RESPIRATORY ALLERGY: A SYSTEMATIC REVIEW OF OBSERVATIONAL COMPARATIVE EFFECTIVENESS STUDIES.

Danilo Di Bona,^1^^ Giovanni Paoletti,^2^^ Derek K. Chu,^3^ Luigi Macchia,^1^ Enrico Heffler,^2^ and Giorgio Walter Canonica.^2^

SUPPLEMENTS

**Methods**

***Search method for the identification of the studies***

The following search strategy was use to search MEDLINE:

#1 Allerg* AND immunotherapy

#2 Specific immunotherapy

#3 AIT

#4 SCIT

#5 SLIT

#6 Subcutaneous immunotherapy

#7 Sublingual immunotherapy

#9 #1 OR #2 OR #3 OR #4 OR #5 OR #6 OR #7 OR #8

#10 Rhin*

#11 Asthma*

#12 #10 OR #11

#13 Long-term

#14 Control*

#15 Real life OR real-life OR real-world

#16 Follow-up

#17 Observational

#18 Prospective

#19 Retrospective

#20 #13 OR #14 OR #15 OR #16 #17 OR #18 OR #19

#21 #9 AND #12 AND #20

#22 Animal

#23 Food allergy

#24 Randomized

#25 Placebo

#26 Double blind

#27 #21 NOT #22 NOT #23 NOT#24 NOT #25 NOT 26

The date of the last search was April 30, 2020.

All other search strategies were modelled on the MEDLINE version.
