# Supplementary material for: Allergen immunotherapy for respiratory allergy: Quality appraisal of observational comparative effectiveness studies using the REal Life Evidence AssessmeNt Tool. An EAACI methodology committee analysis
Source: Clin Transl Allergy. 2021 Jun 14;11(4):e12033. doi: 10.1002/clt2.12033 (PMC8203181; doi:10.1002/clt2.12033)
Supplement: Supplementary file 2 — Supporting Information 2 [file CLT2-11-e12033-s003.docx]

| Study (yr) | Country | Study type | Groups | Participants  over course of study | Female  (%) | Mean Age, yrs  (range) | Sensitization | Asthma (%) | Rhinitis  (%) | Type of AIT | Outcome | Treatment duration  (Yrs)^‡^ | Evaluation  Period |
| --- | --- | --- | --- | --- | --- | --- | --- | --- | --- | --- | --- | --- | --- |
| CHILDREN |  |  |  |  |  |  |  |  |  |  |  |  |  |
| Eng  2002 | Switzerland | P, M | AIT  C | 14 → 13  14 → 10 | 23^˄^  20 ^˄^ | 9.6 (5-16) ^˄^  8.8 (7-13) ^˄^ | Mono/Poly-S | 69  80 | 100^˄^  100^˄^ | SCIT | RSS, MS, SMS | 3 | 6 years after EOT |
| Di Rienzo  2003 | Italy | P | AIT  C | 35 → 35  25 → 25 | 49  48 | 8 (3-17)  9 (4-17) | Mono-Poly-S | 89^˄^  92^˄^ | 100^˄^  100^˄^ | SLIT | ASS, MS | 4 to 5 | 4 to 5 years after EOT |
| Acquistapace  2009 | Italy | P, M | AIT  C | 90 → 81  81 → 81 | 28  27 | 11  12 | Poly-S | 15  24 | 100^˄^  100^˄^ | SLIT | SS, MS | 3 | EOT |
| De Castro  2013 | Italy | P, M | AIT  C | 70 → 64  70 → 63 | 39  47 | 10.4 ± 3  10.7 ± 3 | Mono/Poly-S | 15^˄^  8^˄^ | 86^˄^  91^˄^ | SLIT | RSS, ASS,  MS | 3 | EOT |
| Djuric-Filipovic  2017 | Serbia | P, M | AIT  C | 34 → 34  25 → 25 | -  - | 13.2 ± 3.4 | Mono/Poly-S | -^˄^  -^˄^ | -^˄^  -^˄^ | SLIT | RSS, ASS,  MS, LF, FeNO | 2 | EOT |
| ADULTS |  |  |  |  |  |  |  |  |  |  |  |  |  |
| Arena  2003 | Italy | P | AIT  C | 79 → 71  31 → 63 | 45 | 22.4 - 35.5 | Mono/poly-S | 39.1^˄^ | 57.3^˄^ | SCIT  SLIT | Physician or patient opinion | 3 | EOT |
| Giovannini  2005 | Italy | P, M | AIT  C | 15 → 15  15 → 15 | 47  60 | 24.2  23.9 | Mono -S | 0 | 100 | SCIT | SS, MS | 3 | EOT |
| Marogna  2007 | Italy | R, M | AIT  C | 65 → 53  20 → 12 | 59  40 | 25.6  25.9 | Mono -S | -^˄^  -^˄^ | -^˄^  -^˄^ | SLIT | RSS, ASS,  MS, LF | 3 | 12 after EOT |
| Marogna  2008 | Italy | R | AIT  C | 69 → 57  51 → 44 | -  - | 18-28/55-65  18-28/55-65 | Mono -S | -^˄^  -^˄^ | -^˄^  -^˄^ | SLIT | SMS, LF, drug use | 3 | EOT |
| Milani  2008 | Italy | P, M | AIT  C | 154 → 154  151 → 151 | 52  53 | 22  23 | Mono/poly-S | 0  0 | 100^˄^  100^˄^ | SLIT | RSS, MS | 2 | EOT |
| Dominicus  2012 | Germany | P | AIT  C | 26  13 | 73  54 | 41.7 (24-63)  33.9 (19-47) | Mono/Poly-S | n.r.  n.r. | 100^˄^  100^˄^ | SCIT | SMS, QoL | 3 | 3 years after EOT |
| Drossaert  2016 | France | R, M | AIT  C | 82 → 82  352 → 352 | 59  57 | 33.1 ± 11.6  34.6 ± 12.7 | Mono/Poly-S | 14  12 | 100^˄^  100^˄^ | SCIT | Symptoms,  Medication use | 3 | EOT |
| Bozek  2017 | Poland | P, M | AIT  C | … → 1006  … → 967 | 50  49 | 25.1 ± 9.2  19.7 ± 6.3 | Mono-S | 21  18 | 98^˄^  81^˄^ | SCIT | RSS, ASS,  MS | 3 | 17 years after EOT |
| Rhyou  2020 | Korea | R, M | AIT  C | 56 → 48  75 → 69 | 51  51 | 43.4 ± 12.3  46.9 ± 16.1 | Mono/Poly-S | 100^˄^  100^˄^ | 91.7  60.9 | SCIT | ICS use reduction | 3 | EOT |

Table 1. Characteristics of Studies Assessing the Effects of Allergen Immunotherapy on the Development of New Sensitizations.

Yr, year; n.r., not reported; P, prospective; M, matched; R, retrospective; AIT, allergen immunotherapy; HDM, House Dust Mite; C, controls, subjects not treated with AIT; EOT, end of treatment; FU, follow-up; *, median; →, patients analyzed at the end of the study; ^§^ percentage of males for both groups; D. pt, *Dermatophagoides* *pteronissinus*; D. fa, *Dermatophagoides* *farinae*; ° data available only for AIT; ^**^, discrepancy between data in the text and in the tables in the original studies; data reported in the text are shown here; ^†^ AIT-ad, aluminium hydroxide or calcium phosphate adsorbed extracts; ^‡^ AIT acqueous extracts. ^˄^Disease for which AIT was primarily indicated. Mono-S, mono-sensitized; Poly-S, poly-sensitized; TU, therapeutic units; PNU, protein nitrogen units. RSS, rhinitis symptom score; MS, medication score; SMS, symptom-medication score; ASS, asthma symptom score; LF, lung function; QoL, Quality of Life; ICS, inhaled corticosteroids.
