# Supplementary material for: Allergen immunotherapy for respiratory allergy: Quality appraisal of observational comparative effectiveness studies using the REal Life Evidence AssessmeNt Tool. An EAACI methodology committee analysis
Source: Clin Transl Allergy. 2021 Jun 14;11(4):e12033. doi: 10.1002/clt2.12033 (PMC8203181; doi:10.1002/clt2.12033)
Supplement: Supplementary file 3 — Supporting Information 3 [file CLT2-11-e12033-s002.docx]

Table 2. Summary table of literature analysis

| Reference | Statement | Similar evidence from RCTs | Additional data relative to RCTs |
| --- | --- | --- | --- |
| Acquistapace | A 3-year SLIT course is effective in reducing rhinitis symptoms (RSS) medications (MS), and occurrence new sensitizations. | Yes | New sensitizations |
| Arena | A 3-year SLIT/SCIT course effective in reducing rhinitis symptoms and drug consumption according to physician’s and patient’s opinion, in increasing patient’s satisfaction, and reducing school and work days lost. | Yes | Patient’s satisfaction, working and school days lost |
| Bozek | AIT effectiveness persists after discontinuation (long-term follow-up, 20 years). | No | Effect after discontinuation |
| De Castro | A 3-year SLIT course is effective in reducing rhinitis and asthma symptoms (RSS, ASS) and medications (MS). | Yes | No |
| Di Rienzo | A 3-year SLIT course is effective in reducing asthma development, asthma symptoms (ASS) and new sensitizations (MS). The benefit persists after discontinuation (long-term follow-up, 4 to 5 years) | No | Effect after discontinuation, asthma development, new sensitizations |
| Djuric-Filipovic | A 2-year course of SLIT is effective in reducing rhinitis symptoms (RSS), asthma symptoms (ASS), medications (MS) | Yes | FeNO, FEV_1_ |
| Dominicus | AIT effectiveness persists 3 year after discontinuation (in comparison with AIT untreated controls). | No | Effect after discontinuation, new sensitization, QoL |
| Drossaert | A 3-year SLIT course is effective in reducing symptoms and medication use, as resulted by questionnaires (retrospective assessment). | Yes | No |
| Eng | AIT effectiveness in reducing symptoms and medication use persists after discontinuation (12 years after discontinuation). | No | New sensitization, long-term FU after discontinuation |
| Giovannini | A 3-year SLIT course is effective in reducing rhinitis symptoms (RSS) medications (MS) | Yes | No |
| Marogna ‘07 | AIT effectiveness in reducing symptoms and medication use persists after discontinuation (up to 8 years after discontinuation). | No | Effect after discontinuation |
| Marogna ‘08 | AIT effectiveness in reducing symptoms and medication after 3-years treatment | Yes | Lung function |
| Milani | A 3-year SLIT course is effective in reducing symptoms and medication use | Yes | New sensitizations |
| Rhyou | >1year AIT course reduces ICS in asthmatic patients at 3 years from start of AIT | No | Effect after discontinuation |

RSS, rhinitis symptom score; MS, medication score; ASS, asthma symptom score; FeNO, Fractional exhaled nitric oxide; FEV_1_, Forced Expiratory Volume in 1 second; QoL, Quality of Life.
